# A new treatment for neurogenic inflammation caused by EV71 with CR2-targeted complement inhibitor

**DOI:** 10.1186/1743-422X-9-285

**Published:** 2012-11-23

**Authors:** Shaofu Qiu, Nan Liu, Leili Jia, Guang Yang, Wenli Su, Jing Li, Lixue Song, Chaojie Yang, Jian Wang, Chuanfu Zhang, Zhongqiang Wang, Fei Qiao, Stephen Tomlinson, Carl Atkinson, Yansong Sun, Liuyu Huang, Hongbin Song, Yong Wang, Zhenjun Li

**Affiliations:** 1 Institute of Disease Control and Prevention, Academy of Military Medical Sciences, Beijing 100071, China; 2 Department of Pharmacology, General Hospital of Beijing Military Region, Beijing 100700, China; 3 Department of Microbiology and Immunology, Medical University of South Carolina, Charleston, South Carolina 29425, USA; 4 State Key Laboratory for Infectious Disease Prevention and Control, National Institute for Communicable Diseases Prevention and Control, Chinese Center for Disease Control and Prevention, Beijing, 102206, China

## Abstract

**Background:**

Enterovirus 71 (EV71), one of the most important neurotropic EVs, has caused death and long-term neurological sequelae in hundreds of thousands of young children in the Asia-Pacific region in the past decade. The neurological diseases are attributed to infection by EV71 inducing an extensive peripheral and central nervous system (CNS) inflammatory response with abnormal cytokine production and lymphocyte depletion induced by EV71 infection. In the absence of specific antiviral agents or vaccines, an effective immunosuppressive strategy would be valuable to alleviate the severity of the local inflammation induced by EV71 infection.

**Presentation of the hypothesis:**

The complement system plays a pivotal role in the inflammatory response. Inappropriate or excessive activation of the complement system results in a severe inflammatory reaction or numerous pathological injuries. Previous studies have revealed that EV71 infection can induce complement activation and an inflammatory response of the CNS. CR2-targeted complement inhibition has been proved to be a potential therapeutic strategy for many diseases, such as influenza virus-induced lung tissue injury, postischemic cerebral injury and spinal cord injury. In this paper, a mouse model is proposed to test whether a recombinant fusion protein consisting of CR2 and a region of Crry (CR2-Crry) is able to specifically inhibit the local complement activation induced by EV71 infection, and to observe whether this treatment strategy can alleviate or even cure the neurogenic inflammation.

**Testing the hypothesis:**

CR2-Crry is expressed in CHO cells, and its biological activity is determined by complement inhibition assays. 7-day-old ICR mice are inoculated intracranially with EV71 to duplicate the neurological symptoms. The mice are then divided into two groups, in one of which the mice are treated with CR2-Crry targeted complement inhibitor, and in the other with phosphate-buffered saline. A group of mice deficient in complement C3, the breakdown products of which bind to CR2, are also infected with EV71 virus. The potential bioavailability and efficacy of the targeted complement inhibitor are evaluated by histology, immunofluorescence staining and radiolabeling.

**Implications of the hypothesis:**

CR2-Crry-mediated targeting complement inhibition will alleviate the local inflammation and provide an effective treatment for the severe neurological diseases associated with EV71 infection.

## Background

Enterovirus 71 (EV71) is the major causative agent of hand-foot-and-mouth disease (HFMD) [1]. Since the virtual eradication of the poliovirus, EV71 has been recognized as the most important neurotropic EV. It can cause various neurological diseases, such as aseptic meningitis, acute flaccid paralysis, brainstem encephalitis and fatal neurogenic pulmonary edema [2,3].

Since the first identification of EV71 in 1969, several epidemic outbreaks have been reported in the Asia-Pacific region (Malaysia in 1997, Australia in 1999, Singapore in 2000, Japan in 1997 and 2000, Taiwan in 1998, 2000, 2001 and 2002, and Mainland China in 1998, 2004 and 2008) [3-6]. There were more than 1.1 million HFMD cases including 353 deaths due to the neurological disease in China in 2009 [7]. Mortality was particularly high in EV71-induced brainstem encephalitis complicated with pulmonary edema, especially in children under 5 years of age. EV71 infection has therefore become an important public health problem in the world, particularly in the Asia-Pacific region. EV71 displayed genetic diversity and the virus circulating in this region underwent rapid evolutionary change [8,9], which hampered the development of antiviral agents and vaccines for EV71 infection. As currently no specific antiviral agents or vaccines are available, we should seek a new therapeutic approach to alleviate the severity of EV71-induced neurological diseases.

### Presentation of the hypothesis

#### EV71 is involved in the inflammatory response of the central nervous system

In recent EV71 epidemics in the Asia-Pacific region, the serious complications were mainly associated with the central nervous system (CNS), and the primary lethal symptom was neurogenic pulmonary edema [10]. Magnatic Resonance Imaging and autopsy examinations showed that the pathological lesions occurred predominantly in the brainstem and the spinal cord, rather than in the lung or heart [1,11]. The EV71-associated inflammatory response was found mainly in the CNS region but not in other organs of EV71-infected patients [2,3,12], indicating that the CNS is the major target of EV71 infection. EV71 can enter the CNS through peripheral nerves via retrograde axonal neuronal transmission way or via viremic spread through the blood–brain barrier (BBB). It then induces the human immune cell lines and triggers NF-кь activation to produce proinflammatory cytokines leading to an inflammatory response of the CNS [2,3,10]. Besides, many molecules, such as cyclooxygenase-2 and its metabolite, the cellular protein Cdk5 and others, can facilitate EV71 replication in neural cells and induce neural apoptotic cell death [3]. It is now widely accepted that the extensive peripheral and CNS inflammatory response accompanied by the excessive release of cytokines and chemokines is responsible for the pathogenesis of EV71-associated neurological diseases. These can cause neuronal degeneration, CNS necrosis and destruction of vasomotor function in the brainstem, leading to autonomic nervous system dysregulation and even fatal neurogenic pulmonary edema [13-16]. Patients with brainstem encephalitis and neurogenic pulmonary edema showed elevated levels of inflammatory CNS cytokines, including TNF-α, IL-1β, and IL-6, IL-10, IL-13 and IFN-γ, and a marked depletion of CD4^+^ and CD8^+^ T cells and NK cells [1,11,14], demonstrating the correlation between the extensive CNS inflammatory response and EV71-associated neurological diseases.

#### Inflammatory injury induced by invading pathogens is associated with complement activation

Complement is a key system for immune surveillance and homeostasis, and it bridges the innate and acquired immune responses [17,18]. Under normal circumstances, the immune response recognizes, attacks and eliminates the invading pathogens and this response is beneficial for the host. However, inappropriate or excessive activation of the complement system can result in severe inflammation or tissue injury involved in numerous pathological conditions. Previous studies have noted that many diseases, including the neurological diseases known as transmissible spongiform encephalopathies (TSEs), are due to inflammatory injury. This injury occurs as a result of immune activation by the invading pathogens, instead of being directly induced by the pathogens themselves. TSEs are fatal, neurodegenerative diseases caused by prion protein PrP^Sc^. PrP^Sc^ present in the brain can activate complement, and the deposited complement components C1q, C3b and the membrane-attack complex have all been detected in the brains of patients with prion disease, the pathology of which is due to the propagation of inflammation [19]. It has been proved that an excessive inflammatory reaction in lung tissues induced by influenza virus infection is closely related to complement activation, and complement activation can seriously influence the severity of lung injury [18,20].

The detailed pathogenesis of enteroviruses including EV71 remains unclear, but it has been documented that enterovirus infection can induce an extensive peripheral and CNS inflammatory response [3,11,14]. Our previous results have proved that the complement components C1q and C3 are observed as obvious depositions in the brainstems of mice showing the neurological symptoms of EV71 infection (data not shown), indicating that the inflammatory response in the mouse CNS is associated with complement activation. Anderson *et al.* showed that coxsackievirus B3 (CVB3) capsid proteins could interact with complement C3 and activate the alternative complement pathway after *in vivo* murine infection [21]. The research conducted by Shih *et al.* revealed that EV71 infection could lead to an increased level of mRNAs encoding complement proteins [22]. These results also illustrated the activation of complement due to EV71 infection.

#### Targeted complement inhibition is a potential therapeutic strategy for the inflammatory response

Complement activation can generate inflammatory products including C2a, C3a, C4a and C5a which mediate tissue injury [18,23]. C3a and C5a have the functions of anaphylatoxins and chemoattractants with a wide range of bioactive properties which can induce and increase the inflammatory reaction [24,25]. Complement proteins on cell membranes can be receptors for activated complement proteins or proteins that regulate complement. Complement receptor 2 (CR2), a member of the C3 binding protein family, serves as an important interface between the complement system and adaptive immunity. The specific ligands for CR2 are iC3b, C3dg and C3d, all being cell-bound breakdown fragments of C3 which are deposited on the complement-activating cell surface [24,26,27]. Thus, CR2 is a rational target for the delivery of complement inhibitors such as Crry and CD59 to inflammatory sites that have been induced by complement activation. As Crry has different levels of species selectivity and acts as an important complement regulatory protein, it is considered as an effective complement inhibitor which can regulate complement activation by inhibiting the activity of C3/C5 convertase. CR2-Crry, an ideal targeted inhibitor which has shown increased bioavailability and efficacy and decreased immunosuppression [18,27,28], could provide an appropriate therapeutic strategy for many inflammation-related diseases such as lung tissue injury induced by influenza virus, postischemic cerebral injury and spinal cord injury.

#### Hypothesis

Previous studies indicated that CR2-Crry had an appropriate bioavailability for targeted complement inhibition for the treatment of spinal cord injury and cerebral ischemia/reperfusion injury [27,28]. Our experiments also proved that CR2-Crry could obviously inhibit the pulmonary inflammatory response caused by influenza virus [18]. Thus, targeted complement inhibition induced by CR2-Crry may be an effective therapeutic strategy for EV71 infection of the CNS. Complement activation is a double-edged sword leading to both physiological defense and pathological damage, and systematic complement inhibition may result in potential side effects including infection [25]. Thus, a new treatment strategy for EV71 infection is required. Here, the complement inhibitor CR2-Crry, which targets the sites of complement activation, is proposed as a specific inhibitor of local complement activation without causing severe systemic side effects.

### Testing the hypothesis

The recombinant fusion protein CR2-Crry is prepared by joining the mouse CR2 sequence to the sequence encoding the extracellular region of mouse Crry, and is expressed and purified as described previously [26,29]. The biological activity of the recombinant CR2-Crry is characterized by complement inhibition assays. 7-day-old ICR mice are inoculated intracranially with EV71 BrCr strain to duplicate the neurological symptoms. Then the mice are divided into two groups, in one of which the mice are treated with CR2-Crry, and in the other with phosphate-buffered saline. A group of mice deficient in complement C3 are also each infected with EV71 virus. Animals are perfused transcardially with isotonic saline followed by 4% paraformaldehyde. The brains and spinal cords are either frozen for cryosectioning or processed in paraffin. Immunofluorescence staining is used to assess the presence of complement component C3 and CR2-Crry in the sites of inflammatory, and radiolabeling is conducted to detect the tissue distribution of ^125^I-labeled CR2-Crry in control mice and in mice infected with EV71.

### Implications of the hypothesis

Since the outbreak of HFMD in Shandong and Anhui provinces in 2008, EV71 is gradually spreading to other provinces of China and has become a serious threat to public health in these regions. The EV71-associated neurological diseases which often cause deaths in children under 5 years of age are not directly induced by the virus itself, but are attributable to the inflammation due to EV71 infection. Whilst antiviral therapy is one possible approach, it is perhaps more important to inhibit the inflammatory response caused by EV71. Compared with systemic inhibition, CR2-mediated targeting complement inhibition should greatly reduce the inflammatory reaction or tissue injury resulting from excess complement activation. Thus, verification of the current hypothesis could lead to an effective treatment for EV71 infection.

## Competing interests

The authors declare that they have no competing interests.

## Authors’ contributions

SFQ, NL and LLJ prepared the manuscript. SFQ, NL, LLJ, GY, WLS, LJ, LXS, CJY, JW, CFZ, ZQW, FQ, ST, CA, YSS, LYH, HBS, YW and ZJL participated in developing the hypothesis and collaborated in writing and reviewing of the article. All authors read and approved the final manuscript.
